# Chemotherapy-Induced Neurotoxicity: Molecular Mechanisms, Biomarkers and Emerging Neuroprotective Strategies

**DOI:** 10.3390/biomedicines14081849

**Published:** 2026-08-18

**Authors:** Saahil A. Singh, Rajesh Godvarthi, Prabodh Wankhade, Abhishek Gaurav, Shilpa Narwade

**Affiliations:** 1 Department of Pharmacology, Jawaharlal Nehru Medical College, Datta Meghe Institute of Higher Education and Research, Wardha 442107, Maharashtra, India; godavarthi.ra@gmail.com (R.G.); prabodhw18@gmail.com (P.W.);; 2 Department of Anatomy, Jawaharlal Nehru Medical College, Datta Meghe Institute of Higher Education and Research, Wardha 442107, Maharashtra, India; shilpawp15@gmail.com

**Keywords:** chemotherapy-induced neurotoxicity, chemotherapy-induced peripheral neuropathy, CIPN, chemotherapy-related cognitive impairment, chemobrain, neuroinflammation, mitochondrial dysfunction, *SARM1*, neurofilament light chain, biomarkers, neuroprotection, precision medicine

## Abstract

Chemotherapy-induced neurotoxicity is a major dose-limiting complication of systemic anticancer therapy that adversely affects neurological function, treatment continuity, and long-term quality of life among cancer survivors. Chemotherapy-induced peripheral neuropathy (CIPN) is the most common manifestation, whereas chemotherapy-related cognitive impairment (CRCI) and selected immune-mediated neurological toxicities increasingly contribute to survivorship-related morbidity. Although these immune-mediated syndromes are distinct from conventional chemotherapy-induced neurotoxicity, they are included because they share several downstream pathogenic mechanisms relevant to biomarker discovery and neuroprotective strategies. This structured narrative review critically synthesizes current evidence on the molecular mechanisms, clinical manifestations, biomarkers, and emerging neuroprotective strategies underlying chemotherapy-induced neurotoxicity. A comprehensive PubMed/MEDLINE search was performed, with emphasis on studies published between 2014 and 2026. Evidence from systematic reviews, meta-analyses, clinical guidelines, randomized controlled trials, prospective clinical studies, and high-quality translational research was critically evaluated. Current evidence indicates that chemotherapy-induced neurotoxicity arises through interconnected mechanisms involving oxidative stress, mitochondrial dysfunction, neuroinflammation, calcium dysregulation, blood–brain barrier disruption, and *SARM1*-mediated programmed axonal degeneration. Among emerging biomarkers, neurofilament light chain (NfL) demonstrates the greatest translational potential for early detection and monitoring, while inflammatory cytokines, circulating microRNAs, pharmacogenomic markers, cerebrospinal fluid biomarkers, and advanced neuroimaging may improve individualized risk stratification. Although duloxetine remains the only guideline-recommended pharmacological treatment for established painful CIPN, mechanism-based therapies targeting mitochondrial dysfunction, neuroinflammation, oxidative stress, and *SARM1* signaling, together with structured exercise and biomarker-guided precision medicine, represent promising translational approaches. However, clinical implementation remains limited by incomplete biomarker validation, heterogeneous study methodologies, and the absence of effective disease-modifying neuroprotective therapies, underscoring the need for large biomarker-guided multicenter clinical trials.

## 1. Introduction

Chemotherapy-induced neurotoxicity is one of the most frequent dose-limiting adverse drug reactions associated with systemic anticancer therapy and remains a major cause of long-term neurological morbidity among cancer survivors [1,2]. Neurological complications occur in approximately 60–80% of patients during treatment, with persistent symptoms affecting 30–40% of patients one year after therapy completion [1]. Chemotherapy-induced neurotoxicity encompasses a broad spectrum of neurological complications involving both the peripheral and central nervous systems. The most common manifestation is chemotherapy-induced peripheral neuropathy (CIPN), whereas chemotherapy-related cognitive impairment (CRCI), commonly referred to as “chemobrain,” represents the principal central nervous system manifestation. Less frequently, chemotherapy may also cause autonomic neuropathy, encephalopathy, and leukoencephalopathy [3,4].

CIPN predominantly affects sensory neurons within the dorsal root ganglia and peripheral nerves, resulting in pain, numbness, paresthesia, sensory ataxia, and functional impairment [1,5]. In contrast, CRCI primarily affects higher cognitive functions, including attention, memory, executive function, and processing speed. Reported rates of CRCI vary widely (15–60%), higher prevalence estimates are generally reported in patients receiving high-dose chemotherapy, anthracycline- and taxane-based regimens, and when sensitive neuropsychological testing rather than patient self-report questionnaires is employed [3,4]. Throughout this review, the term chemotherapy-induced neurotoxicity is used as the overarching concept, with CIPN and CRCI discussed as its principal clinical manifestations.

The clinical significance of chemotherapy-induced neurotoxicity extends well beyond reduced quality of life. Severe neurotoxicity frequently necessitates dose reduction, treatment interruption, or premature discontinuation of chemotherapy, potentially compromising oncological outcomes [6]. Chronic CIPN is associated with persistent neuropathic pain, gait impairment, falls, and long-term functional disability, whereas CRCI has been linked to reduced occupational performance, prolonged psychological distress, and diminished overall quality of life [1,3,7]. As cancer survival continues to improve and multimodal treatment strategies become increasingly common, the long-term burden of treatment-related neurological complications has grown substantially. The present review primarily emphasizes evidence published between January 2014 to July 2026, a period that witnessed major advances in the understanding of chemotherapy-induced neurotoxicity, including improved characterization of molecular mechanisms [8], the emergence of translational biomarkers such as neurofilament light chain [9] and evidence-based neuroprotective strategies summarized in the ASCO guideline by [10], together with recent precision medicine approaches for chemotherapy-induced neurotoxicity [11].

Although the primary focus of this review is neurotoxicity induced by conventional cytotoxic chemotherapy, selected immune-mediated neurological toxicities with immune checkpoint inhibitors (ICIs) and chimeric antigen receptor T-cell (CAR-T) therapies are discussed in a separate contextual framework where relevant. These immune-mediated neurological syndromes are not classified as conventional chemotherapy-induced neurotoxicity but are discussed because they share several downstream pathogenic mechanisms including oxidative stress, neuroinflammation, endothelial activation, blood–brain barrier disruption, mitochondrial dysfunction, and axonal injury [1,12,13,14,15]. Examining these overlapping pathways provides valuable translational insight into common biomarkers, shared molecular mechanisms, and emerging neuroprotective strategies that may ultimately improve the prevention, early diagnosis, and management of treatment-related neurological complications across contemporary cancer therapies [12,15].

Accordingly, this structured narrative review critically synthesizes current evidence regarding the molecular mechanisms, biomarkers, clinical manifestations, and emerging neuroprotective strategies underlying chemotherapy-induced neurotoxicity, while clearly distinguishing immune-mediated neurological toxicities associated with ICIs and CAR-T therapies from conventional cytotoxic chemotherapy-induced neurotoxicity and highlighting mechanistic overlap where these insights advance understanding of disease pathogenesis and therapeutic development.

## 2. Literature Search

This review was conducted as a structured narrative review to critically synthesize current evidence on the molecular mechanisms, clinical manifestations, biomarkers, and emerging neuroprotective strategies associated with chemotherapy-induced neurotoxicity. Given the broad scope of the topic and the integration of mechanistic, translational, and clinical evidence, a structured narrative approach was considered more appropriate than a formal systematic review.

A comprehensive literature search was performed using the PubMed/MEDLINE database and updated during manuscript revision, with the final literature search performed on 6 July 2026. PubMed/MEDLINE was selected because of its extensive coverage of peer-reviewed biomedical and clinical literature relevant to oncology, neurology, pharmacology, and translational medicine. The review primarily included publications published from 1 January 2014 to 6 July 2026, a period encompassing major advances in the understanding of chemotherapy-induced neurotoxicity, including the identification of *SARM1*-mediated programmed axonal degeneration, the emergence of neurofilament light chain (NfL) as a promising biomarker, advances in pharmacogenomics and neuroimaging, and the development of novel neuroprotective strategies. Earlier landmark studies were included where necessary to provide historical context and foundational mechanistic insights.

The search strategy combined Medical Subject Headings (MeSH) with free-text keywords using Boolean operators. As this was a structured narrative review rather than a systematic review, the search strategy was refined iteratively during literature screening. A representative PubMed search strategy included combinations of terms such as: (‘chemotherapy-induced neurotoxicity’ OR ‘chemotherapy-induced peripheral neuropathy’ OR CIPN OR ‘chemotherapy-related cognitive impairment’ OR chemobrain) AND (oxidative stress OR mitochondrial dysfunction OR neuroinflammation OR *SARM1* OR neurofilament light chain OR biomarkers OR neuroprotective strategies) together with additional searches for immune checkpoint inhibitors, CAR-T therapy, and immune effector cell-associated neurotoxicity syndrome (ICANS), as appropriate to the topic under review. To improve the completeness of the review, the reference lists of relevant systematic reviews, meta-analyses, clinical practice guidelines, and highly cited articles were manually screened to identify additional studies of direct relevance.

Studies were selected according to their methodological quality, clinical relevance and translational significance. Priority was given to systematic reviews, meta-analyses, international clinical practice guidelines, randomized controlled trials, prospective clinical studies, and high-quality translational research. Experimental studies were included when they provided important mechanistic insights or evaluated emerging therapeutic targets with potential clinical relevance, whereas conference abstracts were generally excluded. An exception was made when conference proceedings represented the only available source of clinically relevant information regarding an emerging therapeutic strategy of direct relevance to the scope of this review. Duplicate publications and studies lacking direct relevance to the objectives of this review were excluded. Literature screening, study selection, and evidence synthesis were performed by the first author according to the predefined objectives of this structured narrative review, while the interpretation, organization, and scientific content were subsequently critically reviewed by all co-authors prior to manuscript finalization.

The qualitative classifications of evidence strength and translational maturity presented in the summary tables were developed by the authors to facilitate interpretation of the literature and do not represent a formal evidence-grading system. Evidence strength was assessed according to the consistency of published findings, methodological quality, and level of clinical validation. Studies supported by multiple high-quality preclinical investigations together with prospective clinical studies or clinical guidelines were considered to provide strong evidence, whereas findings supported by limited clinical studies or heterogeneous results were classified as moderate. Mechanisms or therapeutic approaches supported predominantly by preclinical studies or preliminary early-phase clinical investigations were classified as emerging. Translational maturity reflected the extent of clinical implementation, ranging from established mechanisms or interventions with clinical application to emerging therapeutic targets that remain under preclinical or early clinical investigation.

## 3. Pathophysiology of Chemotherapy-Induced Neurotoxicity

Chemotherapy-induced neurotoxicity arises from multiple interconnected molecular pathways that collectively contribute to neuronal dysfunction and degeneration. Rather than acting independently, these mechanisms exhibit substantial biological overlap, amplifying neuronal injury through self-perpetuating feed-forward processes. Although the relative contribution of each pathway varies among chemotherapeutic agents, oxidative stress, mitochondrial dysfunction, neuroinflammation, calcium dysregulation, blood–brain barrier (BBB) disruption, and programmed axonal degeneration are consistently implicated across both experimental and clinical studies (Table 1).

**Oxidative stress** represents one of the earliest and best-characterized pathogenic events in chemotherapy-induced neurotoxicity. Numerous chemotherapeutic agents promote excessive generation of reactive oxygen species (ROS), overwhelming endogenous antioxidant defenses and resulting in lipid peroxidation, protein carbonylation, DNA damage, and neuronal dysfunction within dorsal root ganglion (DRG) neurons, which are particularly vulnerable because of their relatively permeable blood–nerve barrier [5,8,17]. Extensive preclinical evidence supports oxidative stress as a central mechanism of neuronal injury; however, clinical trials evaluating antioxidant-based neuroprotective strategies have produced inconsistent results, suggesting that oxidative stress interacts with multiple downstream pathways rather than acting as an isolated pathogenic mechanism.

**Mitochondrial dysfunction** represents another central pathogenic mechanism linking oxidative stress to neuronal degeneration. Chemotherapeutic agents impair mitochondrial bioenergetics, disrupt calcium homeostasis, reduce ATP production, and promote opening of the mitochondrial permeability transition pore (mPTP), ultimately triggering the release of pro-apoptotic factors [5,18,19]. Taxanes primarily interfere with mitochondrial tubulin, whereas platinum compounds induce mitochondrial DNA damage and impair mitochondrial DNA repair [5]. Strong experimental evidence supports the pivotal role of mitochondrial dysfunction; however, despite encouraging preclinical findings, effective mitochondria-targeted therapies have yet to demonstrate consistent clinical benefit.

**Neuroinflammation** has emerged as a major contributor to both the initiation and persistence of chemotherapy-induced neurotoxicity. Taxanes and platinum agents activate Toll-like receptor 4 (TLR4) signaling, promoting macrophage infiltration into the DRG and peripheral nerves, increased production of pro-inflammatory cytokines including tumor necrosis factor-α (TNF-α), interleukin-1β (IL-1β), and interleukin-6 (IL-6), and activation of spinal microglia and astrocytes, resulting in central sensitization and persistent neuropathic pain [20,21,26]. Accumulating translational evidence indicates that acute neuroinflammation is primarily driven by rapid cytokine release and innate immune activation, whereas chronic neuroinflammation is sustained by persistent glial activation, central sensitization, and ongoing cytokine signaling [20,21,26]. However, the complexity of these inflammatory networks has limited the successful translation of anti-inflammatory therapies into routine clinical practice [22].

**Calcium dysregulation** contributes particularly to oxaliplatin-induced neurotoxicity through abnormal activation of transient receptor potential (TRP) channels, including TRPA1, TRPV1, and TRPM8, resulting in neuronal hyperexcitability and the characteristic acute cold allodynia associated with oxaliplatin treatment [8,23]. Although calcium-mediated mechanisms are well established for platinum compounds, their contribution appears to vary among different chemotherapeutic classes, indicating that this pathway may be drug-specific rather than universally applicable.

Programmed axonal degeneration, mediated by sterile alpha and TIR motif-containing protein 1 **(*****SARM1*****)**, has recently emerged as one of the most promising mechanistic discoveries in chemotherapy-induced neurotoxicity. Activation of *SARM1* depletes neuronal nicotinamide adenine dinucleotide (NAD^+^), leading to metabolic collapse and rapid Wallerian-like axonal degeneration [24,27]. Robust preclinical evidence identifies *SARM1* as a key regulator of axonal injury and an attractive therapeutic target. Selective *SARM1* inhibitors have recently entered early clinical development; however, the currently available clinical findings are derived primarily from conference proceedings and should therefore be regarded as preliminary pending publication of full peer-reviewed clinical studies [25]. Accordingly, clinical efficacy in patients with chemotherapy-induced neurotoxicity has not yet been established, and the therapeutic potential of *SARM1* inhibition remains supported predominantly by preclinical evidence [24,25,27].

**Blood–brain barrier (BBB) disruption** appears to play a particularly important role in chemotherapy-related cognitive impairment (CRCI) and immune-mediated neurotoxicity associated with chimeric antigen receptor T-cell (CAR-T) therapy. Cytokine-mediated endothelial activation, disruption of tight junction proteins, and increased BBB permeability facilitate neuroinflammation and central nervous system injury [12,13,15]. Although growing experimental and translational evidence supports BBB dysfunction as an important contributor to central neurotoxicity, its precise clinical significance and therapeutic implications require further investigation.

**Figure 1:**
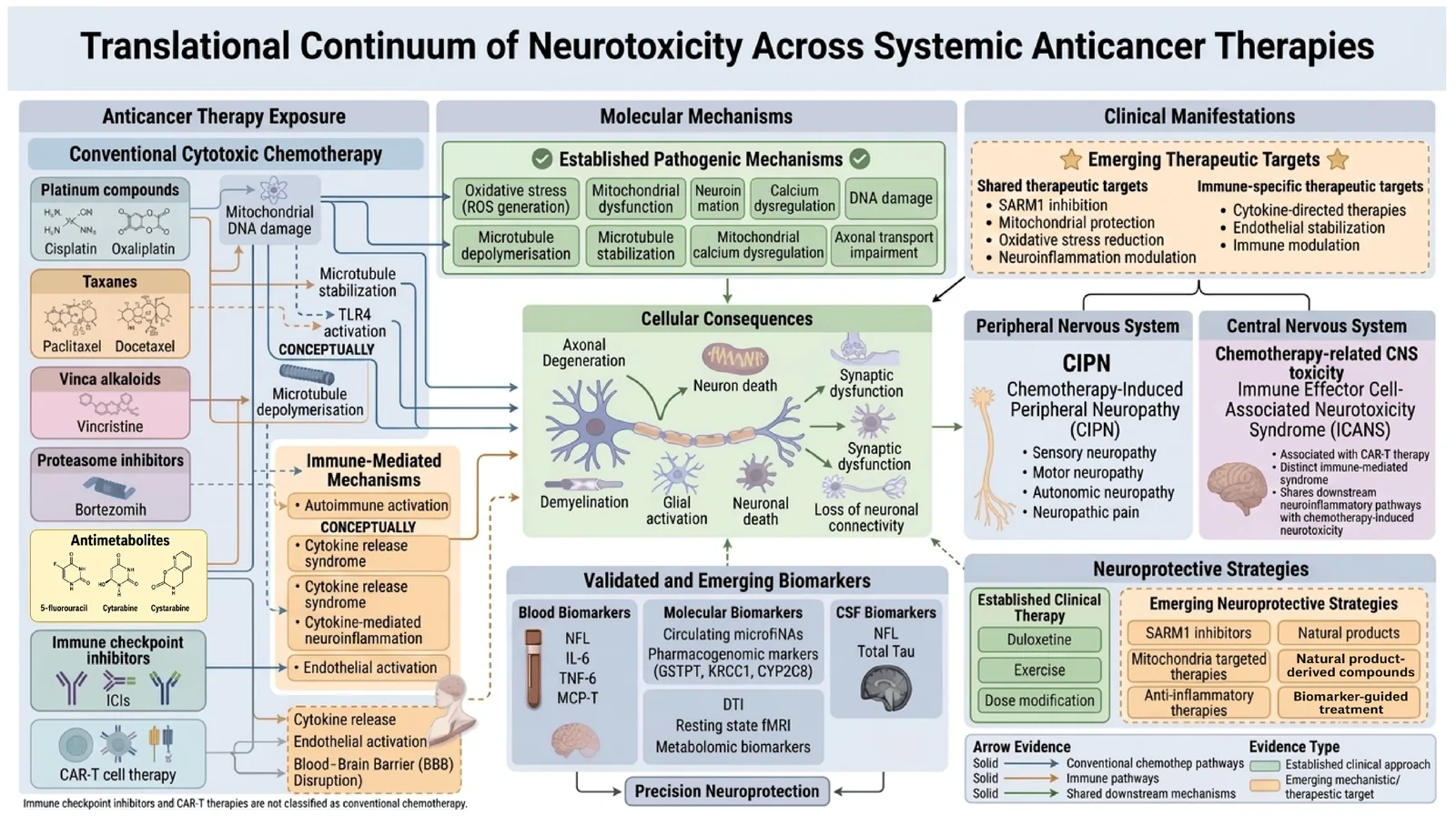
Translational continuum of neurotoxicity across systemic anticancer therapies. Conventional cytotoxic chemotherapy (platinum compounds, taxanes, vinca alkaloids, proteasome inhibitors, and antimetabolites) induces neurotoxicity through mechanisms including oxidative stress, mitochondrial dysfunction, microtubule disruption, calcium dysregulation, DNA damage, and neuroinflammation, resulting in cellular injury, chemotherapy-induced peripheral neuropathy (CIPN), and chemotherapy-related cognitive impairment (CRCI). Immune checkpoint inhibitors (ICIs) and CAR-T cell therapies are not classified as conventional cytotoxic chemotherapy but are included because they produce distinct immune-mediated neurological toxicities while sharing convergent downstream pathogenic pathways, including neuroinflammation, endothelial dysfunction, and neuronal injury, that are relevant to biomarker development and mechanism-based neuroprotective strategies. The figure also summarizes validated and emerging biomarkers and highlights current and investigational neuroprotective approaches, illustrating the translational continuum from molecular mechanisms to clinical manifestations and precision neuroprotection. Solid arrows indicate established mechanistic pathways, whereas dashed arrows represent emerging or investigational pathways.

Ultimately, these interconnected mechanisms converge on neuronal apoptosis, mediated through activation of p53, caspase-3, and BCL-2 signaling pathways, leading to irreversible neuronal loss in susceptible populations [5]. Increasing evidence suggests that chemotherapy-induced neurotoxicity should be viewed as a dynamic, multifactorial process rather than the consequence of a single pathogenic pathway. This integrated understanding has shifted current research toward mechanism-based neuroprotective strategies targeting multiple biological pathways simultaneously and provides the foundation for biomarker-guided precision medicine approaches aimed at preventing or attenuating treatment-related neurological injury [5,8,11,24,27]. Collectively, these interconnected mechanisms form a translational continuum linking systemic anticancer therapy exposure to molecular injury, cellular dysfunction, biomarker development, clinical manifestations, and emerging neuroprotective strategies, as illustrated in Figure 1.

## 4. Drug-Specific Neurotoxic Mechanisms

Although many anticancer agents converge on common pathogenic pathways—including oxidative stress, mitochondrial dysfunction, neuroinflammation, and axonal degeneration—their neurotoxic profiles differ substantially according to drug class, cumulative exposure, and tissue specificity. These mechanistic differences largely determine the onset, severity, reversibility, and clinical manifestations of neurotoxicity, underscoring the need for individualized prevention and management strategies [5,8,22]. Table 2 summarizes the principal classes of anticancer therapies associated with neurotoxicity, including conventional cytotoxic chemotherapies and selected immune-based therapies discussed for comparative mechanistic context, highlighting their dominant molecular mechanisms, characteristic clinical manifestations, dose relationships, major risk factors, and translational relevance.

**Platinum compounds (cisplatin and oxaliplatin)** exhibit preferential accumulation within dorsal root ganglion (DRG) neurons, where they induce DNA adduct formation, mitochondrial dysfunction, and excessive reactive oxygen species (ROS) production [28,29]. Oxaliplatin uniquely causes acute cold-induced dysesthesia through oxalate-mediated hyperexcitability and increased sensitivity of transient receptor potential (TRPA1 and TRPM8) channels, whereas cisplatin more commonly produces cumulative, chronic sensory neuronopathy associated with dorsal column degeneration and persistent glial activation [23,28]. Neurotoxicity is strongly related to cumulative dose and frequently persists long after treatment completion, making platinum-induced peripheral neuropathy one of the most clinically challenging forms of chemotherapy-induced neurotoxicity [29].

**Taxanes (paclitaxel and docetaxel)** disrupt microtubule dynamics, impair axonal transport, and induce mitochondrial injury [5,8]. Paclitaxel additionally activates Toll-like receptor 4 (TLR4), triggering macrophage recruitment and neuroinflammatory signaling that contributes to both acute pain syndromes and chronic chemotherapy-induced peripheral neuropathy (CIPN) [20,21]. Neurotoxicity generally increases with cumulative exposure, although paclitaxel is typically associated with greater neurotoxicity than docetaxel. Persistent glial activation may contribute to the chronicity of symptoms, highlighting neuroimmune signaling as a potential therapeutic target [21].

**Vinca alkaloids**, particularly vincristine, cause neurotoxicity primarily through microtubule depolymerization and disruption of axonal transport [32]. Compared with other chemotherapeutic agents, vincristine more frequently produces early motor and autonomic dysfunction in addition to sensory neuropathy. Neurotoxicity is dose-limiting and often necessitates treatment modification, although partial recovery may occur following dose reduction or discontinuation [28].

**Proteasome inhibitors**, particularly bortezomib, induce endoplasmic reticulum stress, mitochondrial calcium dysregulation, and impaired nuclear factor-kappa B (NF-κB) signaling, resulting predominantly in painful sensory neuropathy. The risk of neurotoxicity is dose-dependent, and modified administration schedules, including subcutaneous delivery and weekly dosing, have reduced but not eliminated this complication. Mitochondrial dysfunction remains an important therapeutic target in this setting [34,35].

**Antimetabolites**, including methotrexate, 5-fluorouracil, and cytarabine, predominantly affect the central nervous system through mechanisms involving impaired hippocampal neurogenesis, oxidative stress, demyelination, and microvascular injury [4,16]. Consequently, these agents are more commonly associated with chemotherapy-related cognitive impairment, leukoencephalopathy, and other central neurotoxic syndromes than with peripheral neuropathy.

The following therapies differ fundamentally from conventional cytotoxic chemotherapy but are included because their neurological toxicities share several convergent molecular pathways relevant to biomarker development and neuroprotective strategies.

**Immune checkpoint inhibitors (ICIs)** produce neurologic immune-related adverse events through loss of immune tolerance and autoimmune activation rather than direct neuronal toxicity [15]. Clinical manifestations include polyradiculoneuropathy, myasthenia gravis-like syndromes, encephalitis, and peripheral neuropathies. Unlike conventional cytotoxic chemotherapy, immune checkpoint inhibitors produce neurological toxicity through immune dysregulation and loss of self-tolerance rather than direct neuronal injury. Consequently, these immune-mediated neurological syndromes are discussed separately from classical chemotherapy-induced neurotoxicity despite sharing several downstream inflammatory mechanisms.

**Chimeric antigen receptor T-cell (CAR-T)** therapy is associated with immune effector cell-associated neurotoxicity syndrome (ICANS), an acute neuroinflammatory complication characterized by cytokine release, endothelial activation, blood–brain barrier disruption, and cerebral edema [12,14]. Although ICANS shares several molecular pathways with chemotherapy-induced neurotoxicity—including neuroinflammation and endothelial dysfunction—it differs substantially in its rapid onset, clinical presentation, and management [12,14]. The severity of ICANS closely correlates with cytokine release syndrome, emphasizing the importance of early recognition and targeted immunomodulatory therapy [12].

Overall, although individual anticancer therapies produce distinct neurotoxic phenotypes, substantial mechanistic convergence exists across oxidative stress, mitochondrial dysfunction, neuroinflammation, calcium dysregulation, and axonal degeneration. These shared biological pathways provide the rationale for mechanism-based neuroprotective strategies, whereas differences in onset, dose dependence, reversibility, and clinical presentation support the development of personalized approaches to prevention and management [5,8,11,29].

## 5. Biomarkers and Diagnostic Assessment of Chemotherapy-Induced Neurotoxicity

### 5.1. Circulating and Molecular Biomarkers

Reliable biomarkers capable of identifying patients at risk of chemotherapy-induced neurotoxicity, facilitating early diagnosis, monitoring disease progression, and objectively evaluating therapeutic response remain an important unmet clinical need. Although numerous candidate biomarkers have been investigated, relatively few have undergone prospective clinical validation. Current evidence suggests that no single biomarker adequately captures the complex and heterogeneous pathophysiology of chemotherapy-induced neurotoxicity, supporting the development of multimodal biomarker-based prediction models [9,11,36]. Table 3 provides a comparative overview of the principal biomarkers and diagnostic assessment modalities currently investigated for chemotherapy-induced neurotoxicity, summarizing their biological significance, potential clinical applications, major limitations, and current translational maturity.

Among the biomarkers currently under investigation, neurofilament light chain has accumulated the strongest clinical evidence and is therefore discussed first.

#### 5.1.1. Neurofilament Light Chain (NfL)

Among currently available candidates, serum neurofilament light chain (NfL) is the most extensively investigated and clinically advanced biomarker. Prospective clinical studies have consistently demonstrated dose-dependent increases in circulating NfL concentrations following treatment with platinum compounds, taxanes, and proteasome inhibitors, with significant correlations between NfL levels and both acute and chronic chemotherapy-induced peripheral neuropathy (CIPN) severity [9,31,37]. Importantly, NfL concentrations may increase before the onset of clinically detectable neurological symptoms, suggesting potential utility for early diagnosis and treatment monitoring.

Prospective studies demonstrate that circulating NfL concentrations increase during chemotherapy and correlate with the severity of chemotherapy-induced peripheral neuropathy. However, the long-term temporal kinetics of NfL after treatment completion remain incompletely characterized. Available longitudinal evidence suggests that persistent NfL elevation may reflect ongoing axonal injury in some patients with chronic neuropathy, although additional prospective studies are required to establish standardized monitoring intervals and clinically validated prognostic thresholds [9,31,37,38].

Despite these promising findings, several important limitations should be recognized. NfL is a general marker of axonal injury rather than a neurotoxicity-specific biomarker, and elevated concentrations may also occur in neurodegenerative disorders, traumatic brain injury, inflammatory neurological diseases, and other conditions associated with neuronal damage. Consequently, interpretation of NfL requires careful consideration of the underlying clinical context. Furthermore, although NfL demonstrates high analytical sensitivity for detecting neuronal injury, its specificity for chemotherapy-induced neurotoxicity remains limited. Standardized diagnostic thresholds, optimal sampling intervals, and clinically validated decision cut-offs have yet to be established. In addition, widespread implementation may be constrained by assay availability, cost, and limited accessibility of ultrasensitive analytical platforms in routine clinical practice. Therefore, NfL should currently be considered a complementary biomarker rather than a replacement for established clinical neurological assessment [9,31,38].

#### 5.1.2. Inflammatory Cytokines

Inflammatory mediators, particularly interleukin-6 (IL-6), tumor necrosis factor-α (TNF-α), and monocyte chemoattractant protein-1 (MCP-1), are consistently elevated in patients with CIPN and have been associated with macrophage infiltration of the dorsal root ganglia, central sensitization, and persistent neuroinflammation [20,21,26]. However, these cytokines lack disease specificity because similar inflammatory profiles occur in malignancy, infection, and other systemic inflammatory conditions, limiting their utility as standalone diagnostic biomarkers.

#### 5.1.3. Circulating microRNAs

Circulating microRNAs (miRNAs), including miR-124, members of the miR-30 family, and miR-100, have emerged as promising regulators of neuroinflammatory signaling, axonal maintenance, and neuronal repair. Differential expression has been reported in both CIPN and chemotherapy-related cognitive impairment (CRCI), suggesting potential utility for early detection and mechanistic stratification [11]. Nevertheless, current evidence is derived predominantly from small exploratory studies, and further multicenter validation is required before clinical implementation.

#### 5.1.4. Pharmacogenomic Biomarkers

Pharmacogenomic biomarkers, including polymorphisms within *GSTP1*, *XRCC1*, and *CYP2C8*, have demonstrated statistically significant associations with platinum- and taxane-induced neurotoxicity in genome-wide association studies [39]. However, individual genetic variants generally confer modest effect sizes and insufficient predictive accuracy for routine clinical decision-making. Future polygenic risk models integrating multiple susceptibility loci may improve individualized risk prediction.

#### 5.1.5. Metabolomic Biomarkers

Metabolomic signatures characterized by alterations in acylcarnitines, sphingolipids, and oxidative stress metabolites provide mechanistic insights into mitochondrial dysfunction and oxidative stress associated with chemotherapy-induced neurotoxicity. Although promising for biomarker discovery and individualized risk prediction, their clinical application remains limited by heterogeneous analytical methodologies, limited prospective validation, and the absence of standardized reference values [9,36].

### 5.2. Cerebrospinal Fluid and Neuroimaging Biomarkers

#### 5.2.1. Cerebrospinal Fluid Biomarkers

Cerebrospinal fluid (CSF) biomarkers, particularly NfL and total tau, provide direct evidence of central nervous system neuronal injury and neurodegeneration and have shown potential utility in the assessment of chemotherapy-related cognitive impairment (CRCI). However, the invasive nature of CSF collection and the limited availability of prospective validation studies currently restrict their routine clinical application [9,36].

#### 5.2.2. Advanced Neuroimaging

Advanced neuroimaging techniques, including diffusion tensor imaging (DTI) and resting-state functional magnetic resonance imaging (rs-fMRI), enable the detection of structural and functional brain alterations associated with CRCI, including white matter injury, hippocampal dysfunction, and altered functional connectivity. Although these approaches provide important mechanistic insights into chemotherapy-related cognitive impairment, their routine clinical application remains limited by cost, technical complexity, lack of standardized imaging protocols, and the absence of validated diagnostic thresholds [9,36].

### 5.3. Clinical Assessment and Integration of Biomarkers

At present, clinical evaluation remains the cornerstone of diagnosis and severity assessment, with validated instruments such as the Common Terminology Criteria for Adverse Events (CTCAE), the European Organisation for Research and Treatment of Cancer Quality of Life Questionnaire–Chemotherapy-Induced Peripheral Neuropathy 20 (EORTC QLQ-CIPN20), and the Total Neuropathy Score (TNS) continuing to guide routine clinical decision-making. Biomarkers such as NfL may improve these assessments by providing objective measures of neuronal injury, but their greatest value is likely to be achieved through integration with standardized clinical assessment tools rather than through independent use [9].

Overall, the greatest future potential lies not in individual biomarkers but in multimodal biomarker panels integrating circulating proteins, inflammatory mediators, microRNAs, pharmacogenomic variants, neuroimaging findings, and standardized clinical assessment tools. Such integrated prediction models are expected to improve risk stratification, facilitate personalized treatment decisions, and enable earlier implementation of neuroprotective interventions. However, clinical implementation of composite biomarker models remains limited by several important challenges, including the high cost and limited accessibility of multimodal assessments, the absence of standardized analytical methods and testing protocols, variability in biomarker performance across different chemotherapeutic agents and treatment regimens, and the lack of clinically validated diagnostic and prognostic thresholds. Consequently, large prospective multicenter studies are still required to establish standardized biomarker panels, validate clinically meaningful thresholds, determine their cost-effectiveness, and facilitate widespread clinical adoption [9,11,36,39].

## 6. Clinical Manifestations

The clinical manifestations of chemotherapy-induced neurotoxicity are heterogeneous and depend on the specific anticancer agent, cumulative drug exposure, and individual patient susceptibility. Peripheral nervous system involvement, particularly chemotherapy-induced peripheral neuropathy (CIPN), represents the most common manifestation of conventional cytotoxic chemotherapy. Central nervous system manifestations include chemotherapy-related cognitive impairment (CRCI) and treatment-related encephalopathy, whereas immune-mediated neurological syndromes such as immune effector cell-associated neurotoxicity syndrome (ICANS) occur in patients receiving cellular immunotherapies and are discussed separately because of their distinct pathogenesis and clinical management despite sharing several mechanistic pathways [1,3,29].

Chemotherapy-induced peripheral neuropathy (CIPN) is typically a length-dependent, predominantly sensory axonal neuropathy characterized by distal paresthesia, numbness, dysesthesia, neuropathic pain, impaired vibration and proprioception, and sensory ataxia producing the classic “stocking-glove” distribution [1,30]. Acute neurotoxicity is most characteristic of oxaliplatin, which frequently produces a transient syndrome of cold-induced paresthesia, jaw tightness, muscle cramps, and pharyngolaryngeal dysesthesia that typically occurs during or shortly after drug infusion and usually resolves within several days [23,28]. In contrast, chronic platinum-induced neuropathy develops progressively with cumulative exposure and may persist long after treatment completion [28,29]. Taxanes commonly produce an acute pain syndrome characterized by diffuse myalgia and arthralgia, followed by chronic distal sensory neuropathy, whereas vincristine frequently causes earlier motor and autonomic dysfunction in addition to sensory impairment [5,20,32]. A clinically important feature of oxaliplatin- and cisplatin-induced neuropathy is the “coasting” phenomenon, in which neurological symptoms continue to worsen for weeks or months after chemotherapy has ceased because of ongoing axonal degeneration [28,29].

Autonomic neuropathy occurs most commonly with vinca alkaloids and proteasome inhibitors and typically presents with orthostatic hypotension, constipation, urinary dysfunction, erectile dysfunction, and impaired gastrointestinal motility. Although less common than sensory neuropathy, autonomic dysfunction may substantially impair quality of life and occasionally necessitate chemotherapy dose modification or discontinuation [2,5,24,32].

Chemotherapy-related cognitive impairment (CRCI), commonly referred to as “chemobrain,” affects approximately 15–60% of patients depending on the treatment regimen, timing of assessment, and cognitive testing methodology [3]. The syndrome is characterized by impairments in attention, working memory, executive function, learning, and processing speed, with many patients experiencing persistent cognitive deficits that interfere with occupational performance and daily functioning [3,4]. Functional neuroimaging studies have demonstrated alterations in hippocampal connectivity, white matter integrity, and default mode network activity, providing biological evidence for chemotherapy-associated central nervous system dysfunction [4,16,36]. Although cognitive impairment often improves following treatment completion, a substantial proportion of cancer survivors continue to experience persistent symptoms for years, emphasizing the need for long-term neurological follow-up [3,4].

More severe central neurotoxic syndromes include chemotherapy-associated encephalopathy, seizures, and posterior reversible encephalopathy syndrome (PRES), which have been reported most commonly with high-dose methotrexate and ifosfamide [12,15]. In contrast, immune effector cell-associated neurotoxicity syndrome (ICANS) is a distinct complication of CAR-T cell therapy that results from cytokine-mediated endothelial activation, blood–brain barrier disruption, and neuroinflammation. Although ICANS shares several mechanistic pathways with chemotherapy-induced neurotoxicity, including neuroinflammation and endothelial dysfunction, it differs substantially in its underlying pathogenesis, clinical presentation, grading, and management [12,13,14,15]. Both chemotherapy-associated central neurotoxicity and ICANS often present acutely and require prompt neurological assessment and specialized supportive management.

Importantly, chemotherapy-induced neurotoxicity extends beyond transient treatment-related adverse effects and has significant long-term implications for cancer survivorship. Persistent neuropathic pain, gait instability, falls, functional disability, cognitive impairment, reduced occupational performance, psychological distress, and diminished health-related quality of life have all been reported in long-term survivors [6,7,10]. Severe neurotoxicity frequently necessitates dose reduction, treatment interruption, or premature discontinuation of potentially life-saving anticancer therapy, potentially compromising oncological outcomes [6,10]. Consequently, early diagnosis, standardized neurological assessment, identification of high-risk patients, and timely implementation of evidence-based neuroprotective and rehabilitative strategies remain central goals of contemporary cancer care [10,15].

## 7. Emerging Neuroprotective Strategies

Current neuroprotective strategies can be broadly categorized into established symptomatic therapies, emerging pharmacological agents, natural product-derived compounds, non-pharmacological interventions, and biomarker-guided precision approaches. However, the level of supporting evidence varies considerably, with only duloxetine currently recommended by international clinical guidelines for established painful chemotherapy-induced peripheral neuropathy (CIPN) [10]. Most other interventions remain at the preclinical or early translational stage, highlighting the persistent gap between mechanistic discoveries and clinically effective neuroprotection [10,22,40]. Consequently, contemporary research has shifted from symptomatic management toward mechanism-based interventions targeting oxidative stress, mitochondrial dysfunction, neuroinflammation, programmed axonal degeneration, and biomarker-guided precision medicine [8,22]. Table 4 provides a comparative overview of the principal neuroprotective strategies investigated for chemotherapy-induced neurotoxicity, highlighting their molecular mechanisms, level of supporting evidence, translational stage, major limitations, and current clinical applicability.

### 7.1. Established Clinical Therapy

The 2020 American Society of Clinical Oncology (ASCO) guideline update concluded that duloxetine remains the only pharmacological intervention with moderate-quality evidence for the treatment of established painful CIPN [10]. Acting through inhibition of serotonin and norepinephrine reuptake, duloxetine enhances descending inhibitory pain pathways and provides modest but clinically meaningful pain relief [10]. Nevertheless, its therapeutic effect is limited to symptomatic management, with no evidence that it prevents neuronal injury, reverses axonal degeneration, or modifies disease progression [10,41]. Consequently, an important unmet need remains for therapies capable of preventing chemotherapy-induced neurotoxicity before irreversible neuronal damage occurs.

### 7.2. Emerging Pharmacological Neuroprotective Strategies

Increasing understanding of the molecular mechanisms underlying chemotherapy-induced neurotoxicity has led to the development of targeted neuroprotective strategies. Among these, calmangafodipir, a manganese superoxide dismutase mimetic, has demonstrated a strong mechanistic rationale by reducing mitochondrial oxidative stress. Although evaluated in the Phase III POLAR trials for oxaliplatin-induced neuropathy, overall clinical efficacy was inconsistent, highlighting the challenges of translating promising preclinical findings into routine clinical practice [48].

Another promising therapeutic target is sterile alpha and TIR motif-containing protein 1 (*SARM1*), the central mediator of programmed Wallerian-like axonal degeneration. Experimental inhibition of *SARM1* preserves axonal integrity across multiple preclinical models of chemotherapy-induced neurotoxicity without compromising antitumour efficacy [24,27]. This therapeutic strategy has recently progressed into early clinical development. Preliminary findings from a conference-reported early-phase clinical study of the selective allosteric *SARM1* inhibitor SIR2501monstrated favorable safety, pharmacokinetic properties, and pharmacodynamic evidence of target engagement in healthy volunteers [25]. However, because these findings are currently available only through conference proceedings rather than a full peer-reviewed publication, they should be regarded as preliminary. Clinical efficacy in patients with chemotherapy-induced neurotoxicity has not yet been established, and adequately powered randomized clinical trials are required before routine clinical implementation can be considered [24,25,27].

Other repurposed pharmacological agents have also demonstrated encouraging but preliminary results. Metformin has shown neuroprotective effects through activation of AMP-activated protein kinase (AMPK), improving mitochondrial bioenergetics and reducing oxidative stress [49]. Current evidence suggests that these AMPK-mediated neuroprotective effects have been demonstrated predominantly in platinum- and taxane-induced neurotoxicity models, whereas evidence supporting efficacy across other neurotoxic chemotherapy classes remains limited. Omega-3 fatty acids may reduce neuroinflammation and stabilize neuronal membranes [50]. Similarly, minocycline has been investigated for its ability to suppress microglial activation and inflammatory cytokine release, although clinical findings have been inconsistent. Overall, these therapies remain investigational because current evidence is derived primarily from small clinical studies or preclinical models, emphasizing the need for adequately powered randomized controlled trials before widespread clinical adoption [22].

### 7.3. Natural Products and Emerging Experimental Therapies

Natural product-derived neuroprotective agents have recently gained considerable attention because of their multitarget effects on oxidative stress, mitochondrial dysfunction, neuroinflammation, and apoptotic signalling pathways [51]. Recent experimental studies have demonstrated promising neuroprotective effects of umbelliferone [51], formononetin [52], thymol nanoparticles [53], troxerutin [54], and polydatin [55] in animal models of chemotherapy-induced peripheral neuropathy and chemotherapy-related cognitive impairment. These compounds consistently modulate key molecular pathways, including Nrf2/HO-1 antioxidant signalling, NF-κB activation, NLRP3 inflammasome signalling, mitochondrial apoptosis, and inflammatory cytokine production, resulting in improved behavioural, electrophysiological, biochemical, and histopathological outcomes.

Importantly, several of these agents—including formononetin, thymol nanoparticles, and troxerutin—have demonstrated neuroprotective activity without significantly reducing the antitumour efficacy of commonly used chemotherapeutic agents in preclinical studies, addressing a major challenge in neuroprotective drug development. Nevertheless, despite their strong mechanistic rationale, these therapies remain largely limited to experimental models [52,53,54]. Their clinical applicability is constrained by limited human evidence, uncertain pharmacokinetics and bioavailability, lack of standardized formulations, and the absence of large prospective clinical trials. Consequently, these agents should currently be regarded as promising investigational therapies rather than established clinical interventions.

### 7.4. Non-Pharmacological Strategies

Among non-pharmacological interventions, structured exercise currently demonstrates the most consistent clinical evidence. Multiple randomized controlled trials and meta-analyses have shown that aerobic and resistance exercise improve neuropathy severity, balance, gait performance, and cognitive outcomes in patients receiving chemotherapy [42,43,56]. Proposed mechanisms include enhanced brain-derived neurotrophic factor (BDNF) signalling [44], stimulation of mitochondrial biogenesis [45], reduction in systemic inflammation [46], and improved neuronal plasticity [47].

Other supportive interventions, including acupuncture, cognitive rehabilitation, neuromodulation, and lifestyle optimization, have demonstrated encouraging preliminary findings but currently lack sufficient high-quality evidence for routine recommendation. Their role is therefore considered complementary to established supportive care.

### 7.5. Precision Medicine and Future Directions

Precision medicine represents one of the most promising future directions for preventing chemotherapy-induced neurotoxicity [11,22,39]. Integration of baseline pharmacogenomic profiling with serial measurement of biomarkers such as neurofilament light chain (NfL), inflammatory cytokines, and circulating microRNAs may enable individualized risk prediction, earlier diagnosis, and personalized neuroprotective interventions [9,11,31,39]. However, standardized biomarker thresholds, prospective validation studies, and biomarker-guided clinical trials remain necessary before these approaches can be incorporated into routine oncology practice [11,22,31].

An equally important translational consideration is preservation of antitumour efficacy. Neuroprotective interventions should ideally protect the nervous system without reducing the cytotoxic effectiveness of chemotherapy [24,49,52]. Future research should therefore prioritize mechanism-based combination therapies, biomarker-guided patient selection, standardized outcome measures, and adequately powered multicentre randomized clinical trials to facilitate successful translation from laboratory research to clinical practice.

## 8. Current Limitations and Research Gaps

Despite substantial advances in understanding the molecular mechanisms underlying chemotherapy-induced neurotoxicity, significant translational challenges remain. Most neuroprotective strategies have demonstrated efficacy primarily in preclinical models, and successful translation into clinical practice has been limited by species-specific differences in neurobiology, immune responses, pharmacokinetics, and chemotherapy exposure [22,40]. Furthermore, many clinical studies are underpowered, include heterogeneous patient populations and chemotherapeutic regimens, employ inconsistent outcome measures, and provide limited long-term follow-up, making comparisons across studies difficult and preventing definitive conclusions regarding neuroprotection versus symptomatic improvement [10,15,22].

Biomarker development also remains incomplete. Although neurofilament light chain (NfL) is currently the most promising biomarker for chemotherapy-induced neurotoxicity, its clinical implementation is limited by uncertain diagnostic thresholds, variable sensitivity across different chemotherapeutic agents, and limited specificity, as elevated NfL reflects generalized axonal injury rather than chemotherapy-specific neurotoxicity [9,11,31]. Future studies should evaluate NfL alongside validated clinical assessment tools, neurophysiological testing, and patient-reported outcome measures to improve diagnostic accuracy and risk stratification.

Another important limitation is the lack of validated personalized prediction model [39]. Clinical risk factors—including age, diabetes mellitus, nutritional status, pre-existing neurological disease, cumulative chemotherapy dose, and pharmacogenomic susceptibility—are known to influence neurotoxicity risk but are rarely integrated into prospective biomarker-driven studies [1,5,30,39]. Similarly, most emerging neuroprotective agents, including phytochemicals and nanotechnology-based approaches, remain at the preclinical or early translational stage, with limited evidence regarding long-term safety, optimal dosing, bioavailability, and potential interactions with anticancer therapies [51,52,53,54,55].

An additional translational concern is the possibility that neuroprotective interventions could inadvertently protect tumour cells alongside neural tissue, thereby reducing the antitumour efficacy of chemotherapy. However, current evidence suggests that this concern remains largely theoretical for most emerging neuroprotective strategies. Preclinical studies of *SARM1* inhibition have demonstrated preservation of axonal integrity without compromising the anticancer activity of vincristine or bortezomib, while recent experimental studies of formononetin likewise reported neuroprotection without significant impairment of oxaliplatin antitumour efficacy [24,27,52]. Nevertheless, these findings require confirmation in adequately powered clinical trials, and preservation of oncological efficacy should remain a primary endpoint during the clinical development of future neuroprotective therapies.

Finally, the neurological complications associated with contemporary cancer treatments, including immune checkpoint inhibitors and CAR-T cell therapies, remain incompletely characterized, and their interactions with conventional chemotherapy require further investigation [12,15,22]. Emerging evidence suggests that these therapies may potentiate neurotoxicity through immune-mediated neuroinflammation, endothelial activation, cytokine release, and blood–brain barrier disruption, potentially exacerbating chemotherapy-induced axonal injury and amplifying pre-existing neurotoxic pathways [12,13,14,15]. Future research should therefore prioritize large multicentre, biomarker-guided randomized clinical trials using standardized outcome measures and integrated molecular, pharmacogenomic, and clinical risk assessment models [11,22,31,39]. Such precision medicine approaches will be essential for developing effective neuroprotective strategies that preserve neurological function without compromising antitumour efficacy [10,11,22,52].

## 9. Future Perspectives

Future advances in the prevention and management of chemotherapy-induced neurotoxicity will depend on the integration of molecular biomarkers, precision medicine, and mechanism-based therapeutic strategies [11,22,39]. Multimodal biomarker panels combining neurofilament light chain (NfL), inflammatory cytokines, circulating microRNAs, pharmacogenomic markers, and advanced neuroimaging are likely to improve early diagnosis, risk stratification, and treatment monitoring [9,11,31,39]. Future biomarker-guided randomized clinical trials should integrate these molecular biomarkers with validated clinical assessment tools, neurophysiological testing, and patient-reported outcome measures to enable individualized neuroprotective strategies and facilitate standardized evaluation across studies [11,22].Several promising therapeutic approaches are expected to progress from experimental research toward clinical application, including selective *SARM1* inhibitors, glia-targeted therapies [21,24,26,27].

Emerging concepts such as senolytic strategies may also represent potential future approaches, as targeting senescent cells or reducing senescence-associated secretory phenotype (SASP)-mediated inflammatory signaling could theoretically attenuate persistent neuroinflammation associated with chemotherapy-induced neurotoxicity [57,58]. However, the role of senolytics in CIPN remains largely investigational and requires further preclinical and clinical validation to establish their therapeutic relevance and safety profile [59]. In parallel, natural product-derived neuroprotective agents demonstrating encouraging preclinical efficacy should undergo rigorous multicentre clinical evaluation to determine their long-term safety, efficacy, and translational potential [51,52,53,54,55].

Future precision oncology approaches should incorporate personalized prediction models that integrate established clinical risk factors—including age, diabetes mellitus, nutritional status, pre-existing neurological disease, cumulative chemotherapy exposure, and genetic susceptibility with longitudinal biomarker monitoring and digital health technologies, including standardized cognitive assessments [11,30,39]. Large multicentre prospective survivorship cohorts with harmonized outcome measures will be essential to validate clinically meaningful endpoints, facilitate regulatory approval, and establish effective neuroprotective strategies that preserve neurological function without compromising antitumour efficacy [10,22,40].

## 10. Conclusions

Chemotherapy-induced neurotoxicity is a complex and multifactorial complication of systemic anticancer therapy that arises through interconnected mechanisms involving oxidative stress, mitochondrial dysfunction, neuroinflammation, calcium dysregulation, blood–brain barrier disruption, and *SARM1*-mediated programmed axonal degeneration. Advances in molecular pharmacology and neurobiology over the past decade have substantially improved understanding of disease pathogenesis and identified several promising therapeutic targets and biomarkers, including neurofilament light chain (NfL), pharmacogenomic markers, and *SARM1*. Nevertheless, no universally effective disease-modifying neuroprotective therapy has yet been established for routine clinical practice. Although this review primarily addresses neurotoxicity associated with conventional cytotoxic chemotherapy, selected immune checkpoint inhibitors and CAR-T cell therapies—while not classified as conventional chemotherapy—were included because comparison of these distinct treatment modalities provides valuable insight into shared pathogenic mechanisms that may inform future biomarker development and neuroprotective strategies across modern cancer therapy.

The persistent translational gap reflects biological heterogeneity, incomplete biomarker validation, inconsistent clinical outcome measures, and the limited translation of encouraging preclinical findings into successful clinical interventions. Previous broadly applied prophylactic strategies, including non-specific antioxidant therapies and generalized neurotrophic factor-based interventions, have demonstrated limited or inconsistent clinical benefit, underscoring the need for biomarker-guided, mechanism-specific neuroprotective approaches. Although several emerging pharmacological agents, natural product-derived compounds, and non-pharmacological strategies have demonstrated promising neuroprotective effects in experimental and early clinical studies, most remain investigational and require rigorous prospective validation to establish long-term safety, efficacy, and preservation of antitumour activity.

Future progress will depend on integrating validated molecular biomarkers, pharmacogenomic profiling, clinical risk factors, and standardized clinical assessment tools into biomarker-guided precision medicine approaches. Large multicentre randomized clinical trials employing harmonized outcome measures and mechanism-based patient stratification will be essential to accelerate clinical translation. Rigorous safety evaluation should also be regarded as a prerequisite for future biomarker-guided and mechanism-based neuroprotective strategies to ensure that protection of the nervous system does not inadvertently confer protection to tumour cells or compromise the anticancer efficacy of chemotherapy. Such precision neuroprotective strategies have the potential to reduce the neurological burden of cancer therapy while preserving oncological efficacy, ultimately improving both survival and long-term quality of life for patients with cancer.

## Figures and Tables

**Table 1:** Summary of the principal pathogenic mechanisms underlying chemotherapy-induced neurotoxicity, their biological effects, strength of supporting evidence, and translational maturity.

Mechanism	Principal Biological Effect	Evidence Strength	Translational Maturity	Reference
Oxidative stress	ROS-mediated neuronal injury	Strong preclinical + moderate clinical	Established mechanism	[8,16,17]
Mitochondrial dysfunction	Bioenergetic failure and apoptosis	Strong preclinical + moderate clinical	Established mechanism	[5,17,18]
Neuroinflammation	Cytokine-mediated neuronal injury	Strong preclinical + moderate clinical	Established mechanism	[19,20,21]
Calcium dysregulation	Neuronal hyperexcitability	Moderate, drug-specific	Established for platinum agents	[8,22]
*SARM1* activation	Programmed axonal degeneration	Strong preclinical; preliminary early clinical evidence	Emerging therapeutic target	[23,24,25]
BBB disruption	Central neurotoxicity and CRCI	Moderate translational evidence	Emerging mechanism	[12,13,15]

**Table 2:** Comparative overview of conventional cytotoxic chemotherapies and selected immune-based anticancer therapies associated with neurotoxicity, highlighting their dominant molecular mechanisms, characteristic clinical manifestations, dose relationships, major risk factors, and translational relevance.

Therapy	Therapeutic CLASS	Dominant Molecular Mechanisms	Acute Neurotoxicity	Chronic Neurotoxicity	Dose Relationship	Major Clinical Phenotype	Major Risk Factors	Translational Relevance
Platinum compounds (cisplatin, oxaliplatin)	Conventional chemotherapy	Mitochondrial DNA damage, ROS generation, DRG accumulation [8,17,28,29]; TRPA1/TRPM8 activation [23]	Oxaliplatin: acute cold-induced dysesthesia and paresthesia [23,28]	Persistent sensory axonal neuropathy, coasting phenomenon [28,29,30]	Strong cumulative dose dependence [6,28,30]	Distal sensory neuropathy (CIPN) [1,28,29]	High cumulative dose, pre-existing neuropathy, diabetes, older age, renal dysfunction [6,30]	Strong evidence supporting biomarker development (e.g., NfL) and neuroprotective interventions [9,10,11,31]
Taxanes (paclitaxel, docetaxel)	Conventional chemotherapy	Microtubule stabilization, impaired axonal transport, mitochondrial dysfunction [5,8,19]; TLR4-mediated neuroinflammation [20,26]	Paclitaxel-associated acute pain syndrome and sensory symptoms [5,20]	Chronic painful sensory neuropathy [5,8,22]	Primarily cumulative dose-dependent (greater with paclitaxel than docetaxel) [6,29]	Painful distal sensory neuropathy [5,9]	Previous neuropathy, diabetes, cumulative exposure [6]	Neuroimmune signalling and glial activation represent promising therapeutic targets [20,22,26]
Vinca alkaloids (vincristine)	Conventional chemotherapy	Microtubule depolymerization, impaired axonal transport [24,29]	Early sensory, motor and autonomic dysfunction [32,33]	Mixed sensorimotor neuropathy with autonomic involvement [32]	Dose-limiting toxicity [32]	Peripheral neuropathy with prominent motor and autonomic manifestations [32,33]	Cumulative dose, hereditary neuropathies (e.g., Charcot–Marie–Tooth disease), hepatic dysfunction [32]	Highlights importance of neurological monitoring and early dose modification [32]
Proteasome inhibitors (bortezomib; carfilzomib, ixazomib less commonly)	Conventional chemotherapy	Endoplasmic reticulum stress, mitochondrial calcium dysregulation, NF-κB dysfunction [34,35]	Painful sensory neuropathy during treatment [24,34]	Partial recovery after dose modification; persistent neuropathy may occur [34]	Dose dependent; reduced with weekly subcutaneous bortezomib [34]	Painful distal sensory neuropathy [24,34]	Previous neuropathy, cumulative exposure [34]	Supports investigation of mitochondria-targeted neuroprotective therapies; newer proteasome inhibitors demonstrate lower neurotoxicity [34]
Antimetabolites (methotrexate, 5-fluorouracil, cytarabine)	Conventional chemotherapy	Impaired hippocampal neurogenesis, oxidative stress, demyelination, microvascular injury [4,16]	Acute encephalopathy (selected agents) [4,16]	Cognitive impairment, leukoencephalopathy [3,4,16,36]	Variable according to agent and cumulative exposure [3,16]	Predominantly CNS toxicity (CRCI) [3,4]	High-dose therapy, cranial irradiation, extremes of age [3,4]	Greater relevance to CRCI than peripheral neuropathy; highlights need for cognitive monitoring [3,16]
Immune checkpoint inhibitors (ICIs)	Cancer immunotherapy	Autoimmune T-cell activation, cytokine-mediated neuroinflammation [15]	Immune-related neurological syndromes (encephalitis, myasthenia gravis-like syndrome, polyradiculoneuropathy [15]	Variable; may persist without prompt immunosuppression [15]	Not cumulative dose dependent [15]	Central and peripheral immune-mediated neurotoxicity [15]	Pre-existing autoimmune disease, combination immunotherapy [15]	Requires early recognition and immunomodulatory therapy; mechanistically distinct from classical CIPN [15]
CAR-T cell therapy	Cellular immunotherapy	Cytokine release, endothelial activation, BBB disruption, neuroinflammation [12,13,14]	ICANS, encephalopathy, aphasia, seizures [12,14]	Usually reversible; persistent cognitive deficits may occur in severe cases [14]	Correlates with cytokine release syndrome rather than cumulative dose [12,14]	Acute immune-mediated CNS neurotoxicity [12,14]	High tumour burden, severe CRS, elevated inflammatory biomarkers [12,14]	Demonstrates mechanistic overlap with chemotherapy-induced neuroinflammation while requiring distinct immunomodulatory management [13,14]

**Table 3:** Comparative overview of biomarkers and diagnostic assessment modalities investigated for chemotherapy-induced neurotoxicity, highlighting their biological significance, potential clinical applications, major limitations, and current translational maturity.

Biomarker	Principal Biological Significance	Potential Clinical Utility	Major Limitations	Current Clinical and Translational Maturity	Ref.
Neurofilament light chain (NfL)	Sensitive marker of axonal injury released following neuronal damage	Early detection of CIPN, monitoring disease progression, objective assessment of treatment response, and risk stratification	Not specific to chemotherapy-induced neurotoxicity; elevated in numerous neurological disorders; standardized diagnostic cut-offs lacking; assay availability and cost remain limiting factors	High—the most extensively clinically validated biomarker; prospective studies support its utility, although routine implementation requires further standardization	[9,31,37,38]
Inflammatory cytokines (IL-6, TNF-α, MCP-1)	Reflect systemic and neuroinflammatory activation associated with neuronal injury and glial activation	Identification of inflammatory activity, disease monitoring, and potential pharmacodynamic biomarkers for anti-inflammatory therapies	Poor disease specificity; influenced by malignancy, infection, and other inflammatory conditions; considerable inter-patient variability	Moderate—useful as adjunctive biomarkers but insufficient as standalone diagnostic markers	[11,20,21,26]
Circulating microRNAs (miR-124, miR-30 family, miR-100)	Regulators of neuroinflammation, axonal maintenance, neuronal repair, and gene expression	Early diagnosis, mechanistic stratification, prediction of neurotoxicity risk, and precision medicine approaches	Small study populations, inconsistent findings, lack of standardized analytical methods, and limited external validation	Emerging—promising biomarkers but currently investigational	[11]
Pharmacogenomic biomarkers (*GSTP1*, *XRCC1*, *CYP2C8*)	Reflect inherited genetic susceptibility to chemotherapy-induced neurotoxicity	Identification of high-risk patients before chemotherapy and individualized treatment planning	Individual variants confer modest predictive value; inconsistent replication across populations; insufficient predictive accuracy for routine clinical use	Emerging—may contribute to future polygenic risk prediction models	[30,39]
Metabolomic biomarkers (acylcarnitines, sphingolipids, oxidative stress metabolites)	Reflect disturbances in cellular metabolism, mitochondrial dysfunction, and oxidative stress	Mechanistic insight, biomarker discovery, individualized risk prediction, and identification of therapeutic targets	Limited prospective validation, heterogeneous analytical methodologies, and absence of standardized reference values	Emerging—promising for future precision medicine but currently exploratory	[9,36]
CSF biomarkers (NfL, total tau)	Reflect central nervous system neuronal injury and neurodegeneration	Assessment of chemotherapy-related cognitive impairment (CRCI) and central neurotoxicity	Invasive sample collection limits routine clinical application; limited prospective validation	Moderate—primarily research applications with potential translational value	[9,36]
Advanced neuroimaging (DTI, rs-fMRI)	Detects structural and functional brain alterations associated with CRCI	Evaluation of white matter integrity, hippocampal injury, functional connectivity, and cognitive dysfunction	High cost, limited accessibility, lack of standardized imaging protocols, and uncertain clinical thresholds	Moderate—valuable research tool with limited routine clinical implementation	[36]

**Table 4:** Comparative overview of current and emerging neuroprotective strategies for chemotherapy-induced neurotoxicity, highlighting their principal molecular mechanisms, level of supporting evidence, translational stage, major limitations, and clinical applicability.

Neuroprotective Strategy	Principal Molecular Mechanism	Highest Level of Supporting Evidence	Translational Stage	Major Limitations	Supporting References
Duloxetine	Central serotonergic and noradrenergic modulation	Phase III randomized controlled trial; ASCO guideline	Clinical practice	Symptomatic treatment only; does not prevent axonal degeneration	[10,41]
Exercise (aerobic/resistance)	BDNF upregulation, mitochondrial biogenesis, anti-inflammatory effects, enhanced neuroplasticity	Meta-analysis of RCTs and multicenter RCT	Clinical practice	Variable exercise protocols and adherence	[42,43,44,45,46,47]
Calmangafodipir	Manganese superoxide dismutase (MnSOD) mimetic; mitochondrial ROS scavenging	Phase III randomized clinical trials (POLAR-A/POLAR-M)	Late-stage clinical	Mixed efficacy; clinical development limited following Phase III results	[48]
Metformin	AMPK activation, mitochondrial protection, reduction in oxidative stress	Randomized controlled clinical trial	Early clinical	Limited randomized evidence; requires larger multicenter studies	[49]
Omega-3 fatty acids	Anti-inflammatory activity, neuronal membrane stabilization, reduction in oxidative stress	Randomized double-blind placebo-controlled clinical trial	Early clinical	Limited number of clinical trials; heterogeneous patient populations and dosing regimens	[49,50]
*SARM1* inhibitors (e.g., *SIR2501*)	Prevention of NAD^+^ depletion and programmed axonal degeneration	Preliminary conference-reported Phase I clinical findings supported by extensive preclinical studies	Early clinical development	Clinical efficacy has not been established; currently available human data are limited to conference proceedings, and long-term safety and efficacy require confirmation in peer-reviewed clinical studies and randomized trials.	[24,25,27]
Umbelliferone	NF-κB inhibition, PPAR-δ modulation, regulation of mitochondrial apoptosis	Preclinical animal studies	Preclinical	No clinical trials	[51]
Formononetin	Activation of Nrf2/HO-1 antioxidant pathway; reduction in oxidative stress	Preclinical cell and animal studies	Preclinical	Limited translational validation despite preservation of anticancer efficacy	[52]
Thymol nanoparticles	Activation of Nrf2/HO-1 pathway; attenuation of endoplasmic reticulum stress; inhibition of NLRP3 inflammasome	Preclinical animal studies	Preclinical	No human clinical studies	[53]
Troxerutin	Modulation of gut–brain axis; activation of Nrf2/HO-1 signalling; inhibition of NLRP3 inflammasome	Preclinical animal studies	Preclinical	Absence of clinical validation	[54]
Polydatin	Antioxidant, anti-inflammatory and anti-apoptotic mechanisms	Preclinical animal studies	Preclinical	Limited human evidence	[55]
Biomarker-guided precision neuroprotection	Integration of pharmacogenomics with serial biomarker monitoring (NfL, cytokines, miRNAs)	Prospective observational studies	Emerging	Requires prospective validation, standardized biomarker thresholds and cost-effectiveness analyses	[11,22,31,39]

## Data Availability

No new data were created or analyzed in this study. Data sharing is not applicable to this article.
